# Diversity and putative metabolic function of prokaryotic communities in tank bromeliads along an elevation gradient in tropical Mexico

**DOI:** 10.3389/fmicb.2022.945488

**Published:** 2022-10-13

**Authors:** Yonatan Aguilar-Cruz, Felix Milke, Janina Leinberger, Anja Poehlein, Gerhard Zotz, Thorsten Brinkhoff

**Affiliations:** ^1^Functional Ecology of Plants, Institute of Biology and Environmental Sciences, Carl von Ossietzky University of Oldenburg, Oldenburg, Germany; ^2^Institute for Chemistry and Biology of the Marine Environment, Carl von Ossietzky University of Oldenburg, Oldenburg, Germany; ^3^Department of Genomic and Applied Microbiology, Institute of Microbiology and Genetics, Georg-August University of Göttingen, Göttingen, Germany

**Keywords:** epiphytes, nutrient cycling, metabarcoding, microbiome, microecosystems, biogeography

## Abstract

Tank bromeliads are unique canopy microhabitats that offer freshwater and organic nutrient-rich substrates in the Neotropics. In them it is possible to thoroughly characterize environmental factors and species composition of terrestrial and aquatic biota. Therefore, these plants have been used as natural models to study how communities are distributed and assembled. Here we used amplicon sequencing of the 16S rRNA gene and their functional annotations to study the diversity and metabolic potential of prokaryotic communities in tank bromeliads in five different forests along an elevation gradient in tropical Mexico. Furthermore, we analyzed the effects of vegetation type and environmental factors inside the tanks on prokaryotic composition. We found a high prokaryotic diversity in tank bromeliads along the elevation gradient. Prokaryotes commonly observed in acidic environments rich in organic carbon, and the potential pathogen *Pasteurella multocida*, were present in all samples, but few amplicon sequence variants were shared between forests. The prokaryotic composition was affected by forest type, and comparisons against null models suggest that it was shaped by non-neutral processes. Furthermore, prokaryotic community changes significantly covaried with tank water temperature, pH, and inorganic carbon. We found a high diversity of putative metabolic groups dominated by chemoheterotrophs and fermenters, but taxonomic groups involved in nitrogen and sulfur cycling were also present in all samples. These results suggest that tank bromeliads promote taxonomic and metabolic diversity of the prokaryotic community at a local and regional scale and play an important role in the biogeochemistry of forest canopies in the Neotropics.

## Introduction

Among Magnoliidae, Bromeliaceae are second, only after Orchidaceae, in terms of epiphyte richness (*ca*. 1,800 species, representing 60% of this family), and this family is also the most abundant in many Neotropical forest canopies (Benzing, 2000; Zotz, 2013; Zotz et al., 2021). An outstanding feature of many members of Bromeliaceae (tank bromeliads) is their capacity to capture and store water and litter in tanks formed by their overlapping leaf bases (Benzing, 2000; Zotz et al., 2020; Aguilar-Cruz et al., 2022). Litter trapped by these plants is subsequently decomposed by associated biota, releasing nutrients that can be absorbed directly from the tank solution *via* foliar trichomes (Benzing, 1970). Because of these characteristics, tank bromeliads are considered aerial wetlands that offer freshwater and organic nutrient-rich substrates in the treetops of humid forests of the Americas (Picado, 1913; Paoletti et al., 1991; Zotz and Thomas, 1999; Zotz et al., 2020). Thus, these plants represent unique canopy habitats that can be used by biota in different ways, e.g., their phytotelmata (small bodies of water held by plants) are exploited by aquatic organisms, axils filled with organic matter are occupied by soil fauna, and the tanks are occasionally used as nests, shelters, water source, or foraging grounds by terrestrial visitors (Frank and Lounibos, 2009).

Bromeliad biota have fascinated researchers for decades (Kitching, 2004). They have been extensively studied to understand how communities are assembled by treating bromeliad tanks as small discrete microcosms, where it is possible to both accurately characterize environmental factors and species composition (Armbruster et al., 2002; Dézerald et al., 2014). Information on associated organisms including protozoa, gastrotrichs, rotifers, nematodes, oligochaetes, copepods, ostracods, crabs, insects, and vertebrates can be found in numerous publications, e.g., Picado (1913); Laessle (1961); Benzing (2000); Kitching (2004); Frank and Lounibos (2009); Zotz and Traunspurger (2016). However, associated microbes used to be virtually unknown, but with the advent of next-generation sequencing technology in the last two decades, diverse microbial communities have been described. These communities are distinct from the surrounding environment and present a remarkable variation among and within bromeliad species (Goffredi et al., 2011; Carmo et al., 2014; Louca et al., 2017b; Simão et al., 2020). For example, an analysis of 16S rRNA and 18S rRNA genes showed that communities of bacteria and fungi differed between terrestrial soil and litter accumulated in the leaf axils of epiphytic tank bromeliads, with lower bacterial cell numbers in the terrestrial habitat (Pittl et al., 2010). Other studies based on amplicon sequencing of 16S rRNA genes revealed a high diversity of bacteria inhabiting these plants, with Proteobacteria, Acidobacteria, Bacteroidetes, Verrucomicrobia, and Firmicutes as dominant groups (Louca et al., 2017a; Giongo et al., 2019; Simão et al., 2020). Furthermore, it has been shown that an acidic environment in bromeliad tanks favored the abundance of Acidobacteria and Alphaproteobacteria (similar to those found in acidic, water-logged and peat bog habitats), while Betaproteobacteria and Firmicutes dominated in bromeliads with higher tank pH (Goffredi et al., 2011).

Microbes are not only an important diversity component of the bromeliad associated biota, they also are expected to be functionally crucial for the carbon and nutrient cycling that take place in bromeliad microecosystems (Louca et al., 2017a). Pioneering work on this topic includes the research of Bermudes and Benzing (1991) who found that Ecuadorean bromeliads are sites of nitrogen–fixation, and that of Brighigna et al. (1992) who isolated nitrogen–fixing bacteria from the phyllosphere of 12 species of *Tillandsia* from Mexico. More recently, Inselsbacher et al. (2007) demonstrated an important role of microbes in transforming N compounds in the tank itself, while Giongo et al. (2013) showed that bacteria inhabiting tank bromeliads can solubilize phosphate and produce growth-promoting agents, e.g., siderophores (high-affinity iron-chelating compounds) and the plant hormone indole-3-acetic acid. Finally, archaeal and bacterial 16S rRNA genes and metagenomic functional profiles revealed the presence of a rich repertoire of genes and metabolic functional groups within bromeliad tanks, associated with catabolic pathways for the degradation of organic molecules especially under low oxygen conditions (Martinson et al., 2010; Goffredi et al., 2011; Louca et al., 2017a,b).

Although the study of prokaryotic communities in tank bromeliads has gained considerable momentum in the last decade, our understanding of the composition and the functional diversity of these communities is still very poor (Giongo et al., 2019). Until now, bacteria and archaea have been studied mainly in restinga vegetation and Atlantic, lowland, rain and tropical montane forests (Martinson et al., 2010; Pittl et al., 2010; Goffredi et al., 2011; Carmo et al., 2014; Lehours et al., 2016; Louca et al., 2017b; Simão et al., 2020). However, there is still a lack of information for other common ecosystems of the Neotropics, such as seasonally dry tropical forests and mangroves, where tank bromeliads can also be abundant (De Sousa and Colpo, 2017; Aguilar-Cruz et al., 2022). Moreover, although it is well known that prokaryotic communities differ among and within bromeliad species, comparisons of these communities between ecosystems at a regional scale are still lacking. Such comparisons are important to understand how this high taxonomic variability is shaped and maintained, and to disentangle prokaryotic variability within from that of between vegetation zones. This study closes these research gaps by using amplicon sequencing of 16S rRNA genes and functional annotations to (i) characterize and compare the prokaryotic communities (bacteria and archaea) in tank bromeliads in five different forests along an elevation gradient in tropical Mexico, (ii) analyze the effects of vegetation type and environmental factors (physicochemical parameters of tank water) on the composition of prokaryotic communities in tank bromeliads, and (iii) associate the detected taxa with metabolic functions of ecological importance, with particular emphasis on nutrient and carbon cycling.

## Materials and Methods

### Sampling

Sampling was performed in five different forests located along an elevation gradient formed by the eastern slopes of the Cofre de Perote volcano in central Veracruz, Mexico. This elevation gradient is in a region considered a diversity hotspot in the Neotropics and is characterized by a complex topography and a high diversity of climates, soils, and plant communities, which have been classified into more than 27 elevational vegetation zones (Cházaro-Basáñez, 1992; Carvajal-Hernández et al., 2020). The selected forests along this gradient correspond to a mangrove [MF, 5 meters above sea level (m a.s.l.), mean annual temperature = 25.9°C], a semi-deciduous tropical forest (SDTF, 650 m a.s.l., 22.2°C), a tropical oak forest (TOF, 1,005 m a.s.l., 20.1°C), and two different cloud forests (*CF*-1600, 1,650 m a.s.l., 16.4°C and *CF*-2200, 2,210 m a.s.l., 13.1°C). Further descriptions of the study sites and their epiphyte flora can be found in Aguilar-Cruz et al. (2020, 2021); Guzmán-Jacob et al. (2020).

We sampled three bromeliad specimens in each forest except in the mangrove, where we sampled five (*n* = 17). These belonged to six species common at the study sites (*Aechmea bracteata*, *A*. *nudicaulis*, *Tillandsia heterophylla*, *T*. *imperialis*, *T*. *limbata*, and *T*. *macropetala*). Samples were collected at the end of the rainy season during days without heavy rain (October 10-19th, 2017). Only large individuals (foliar length > 30 cm) located on the trunk or inner branches of trees were selected, and 40 to 50 ml water of each plant were taken from the interfoliar tanks by siphoning it through a plastic tube attached to a syringe. Immediately after the extraction, the tank water was poured into a plastic bottle, its temperature, pH, salinity and dissolved oxygen (OD) were measured five times at 2 min intervals using a multiparameter instrument (model HI98194, Hanna Instruments Inc., Rhode Island, United States). Water samples were transferred into sterilized plastic bottles (further processing is described below). Additionally, sediment and water of each plant were mixed and subsequently collected from the bottom of the leaf axils through plastic tube attached to a syringe. Excess water was removed and about 0.1 ml of the sediments was placed in 2 ml Eppendorf tubes and fixed adding 900 μl of DNA/RNA Shield™. Sediment and water samples were stored in a cooler at 0°C and transported to the laboratory of the Instituto de Ecología A.C. within 24 h. After each sampling, all materials were carefully cleaned by rinsing them several times with distilled water and once with ethanol (70%), in addition they were daily washed and sterilized in distilled boiling water.

### Water chemical analysis

For chemical analysis, 20 ml of the water samples were filtered through a 0.2 μm Whatman® nucleopore track-etch membrane, and the concentration of phosphorous was determined by the colorimetric method with ascorbic acid (AOAC, 1980), using a spectrophotometer (Espectro Max Plus, model 384, Molecular Devices Corporation, Sunnyvale, United States). The remaining unfiltered water samples were sent to the Laboratorio Universitario de Nanotecnología Ambiental (Mexico City) where total nitrogen (N) and carbon (TC) were determined using high temperature platinum catalyzed combustion (TOC-L CSH/CSN Shimadzu, Japan). Total inorganic carbon (IC) was analyzed using the same method, after acidification of the samples with 1 M HCl, and total organic carbon (OC) was calculated as TC-IC.

### DNA extraction and amplicon sequencing

Prokaryotic DNA was extracted from the sediments by the phenol-chloroform method (Giebel et al., 2009). Therefore, 300 μl of sediment of each sample (suspended in DNA/RNA Shield™) were transferred to a 2 ml safe-lock tube to which 0.25 g of combusted zirconium beads were added (Ø = 0.1 mm, BioSpec Products). Then 500 μl of phosphate buffer (pH 8.3), 500 μl of phenol-chloroform-isoamyl alcohol (PCI), and 60 μl of sodium dodecyl sulfate (SDS) 10% were added. Afterward, the tubes were vortexed for 5 min, transferred to a water bath at 60°C for 10 min, and centrifuged at 10,000 rpm for 10 min at 20°C. The supernatants were transferred to clean tubes adding 500 μl of PCI, then vortexed again for 1 min, and centrifuged at 10,000 rpm for 10 min at 20°C. This step was repeated twice until no precipitate appeared in the interphase. Afterward, the DNA was precipitated adding 50 μl Na-acetate (3 M, pH 5.2) and two-fold volume isopropanol (−20°C), then freezing the tubes at −80°C for at least 1 h. Later, the tubes were centrifuged at 13,000 rpm for 30 min at 4°C and the supernatants were carefully decanted. The obtained pellets were washed in 1 ml ice-cold ethanol (80%) and centrifuged at 13,000 rpm for 10 min at 4°C, then the supernatants were decanted again, and the pellets were dried by vacuum centrifugation for *ca*. 5 min. Finally, the pellets were resuspended in 100 μl PCR water and stored at −20°C.

The 16S rRNA genes were amplified for molecular identification of prokaryotic communities (bacteria and archaea) using the broad-range primes 341F: 5′-CCTACGGGNGGCWGCAG-3′, and 785R: 3′-GACTACHVGGGTATCTAATCC-5′ (Klindworth et al., 2013). Settings used in PCR are given in the Supplementary Table S1. Each sample was 1:10 diluted and three individual PCRs were performed to minimize PCR bias. Successful PCR was confirmed by separating reaction products *via* agarose gel electrophoresis and visualizing them under UV light after staining with ethidium bromide. Triplicates of PCR products were pooled and purified using QI Aquick PCR purification kit (Qiagen) and samples were sequenced at the Institute of Microbiology and Genetics (Göttingen, Germany). PCR products were used to attach indices and Illumina sequencing adapters using the Nextera XT Index kit (Illumina, San Diego). Index PCR was performed using 5 μl of template PCR product, 2.5 μl of each index primer, 12.5 μl of 2× KAPA HiFi HotStart ReadyMix and 2.5 μl PCR grade water. Thermal cycling scheme was as follows: 95°C for 3 min, 8 cycles of 30 s at 95°C, 30 s at 55°C and 30 s at 72°C and a final extension at 72°C for 5 min. Quantification of the products was performed using the Quant-iT dsDNA HS assay kit and a Qubit fluorometer (Invitrogen GmbH, Karlsruhe, Germany) following the manufacturer’s instructions. MagSi-NGSPREP Plus Magnetic beads (Steinbrenner Laborsysteme GmbH, Wiesenbach, Germany) were used for purification of the indexed products as recommended by the manufacturer and normalization was performed using the Janus Automated Workstation (Perkin Elmer, Waltham, MA, United States). Sequencing was conducted using the Illumina MiSeq platform using dual indexing and MiSeq reagent kit v3 (600 cycles) as recommended by the manufacturer.

### Bioinformatics

Raw 16S rRNA gene sequences were trimmed to remove primer sequences using cutadapt (Martin, 2013), discarding sequence pairs without primers and with >20% mismatches within the primer sequence. Subsequent steps were conducted with QIIME2 (Bolyen et al., 2019). First, low quality ends of reads were trimmed resulting in read lengths of 250 and 230 bp for forward and reverse, respectively. Further, low quality reads were discarded, and the resulting read pool was denoised using the DADA2 plugin (Callahan et al., 2016). Denoised reads were merged and possible chimeric sequences discarded. Afterward, the resulting amplicon sequence variants (ASVs) were classified using the trained Naïve Bayesian classifier implemented within QIIME2 against a curated SILVA132 database (Quast et al., 2013), which was fitted to the primer region. Plastidal sequences were removed from the dataset and ASV reads were exported into a count table including taxonomical assignments for further statistical analysis. Samples with a low number of reads (<1,000) were discarded (two in the MF and one *CF*-2200).

### Putative metabolic function

We linked metabolic functions to the detected organisms (e.g., genera or species) using the database FAPROTAX, which was designed for marine and lake biogeochemistry, but has also been used to study the functional structure of the tank bromeliad microbiome (Louca et al., 2016, 2017a,b). This database allows the establishment of putative metabolic or ecological functions (e.g., nitrification, methanogenesis, or fermentation) using current literature on cultured strains. For example, if all strains of species within a bacterial genus were identified as nitrifiers it is assumed that all uncultured species of that genus are also nitrifiers (Louca et al., 2016).

Relative abundances of functional groups were calculated considering a total of 1,141 ASVs (23.5% of those taxonomically identified), which were assigned to at least one functional group, and discarding all ASVs that could not be assigned to any group (76.5%). All functional groups contributing less than 1% to the total assignments were grouped into “others.” Core functional groups were visualized by filtering out those groups that were not present in all samples.

### Statistical analysis

Amplicon data were analyzed using R version 4.1.2 (R Core Team, 2020). To visualize community compositions, counts were normalized to proportions and bar plots were produced displaying taxonomy at phylum and genus levels. All phyla and genera contributing less than 1% to the complete dataset were grouped into “others.” Alpha-diversity was calculated after rarefying all samples to a common count number for which we used the minimum of all samples (9,843 counts). Effective number of species was calculated as the inverse Simpson index using the vegan package (Oksanen et al., 2020) and differences between sites were examined using Kruskal-Wallis tests. To characterize the environmental conditions inside the tanks, the physicochemical parameters of bromeliad water were displayed using boxplots and differences in single parameters between forests were tested using Kruskal-Wallis tests. Moreover, a principal component analysis was performed to visualize sample similarity with respect to the environmental conditions.

To present beta-diversity, we first transformed count tables into Bray-Curtis distances and conducted non-metric multidimensional scaling (NMDS) to show variability of communities within the dataset. Afterward, to test the effect of forest type and environmental variables (temperature, salinity, DO, IC, OC, N, and P) on the prokaryotic community composition, we performed individual permutational multivariate analysis of variance (PERMANOVA), using the vegan package. The significance of the pseudo-F statistic was calculated using the Bray-Curtis distance matrix and 10,000 permutations. Moreover, we visualized the distribution of ASVs of selected taxonomic groups by ordering the ASVs according to a hierarchical clustering of their normalized abundance distribution within the dataset. For that, we removed all ASVs that were present in only one sample and the abundance of each ASV was divided by its total sum. Further, optimal number of clusters was inferred by applying the silhouette-clustering approach. Clusters of ASVs were visualized in a heatmap using rows for ASVs and columns for single samples. Rows were ordered and grouped according to the hierarchical clusters, whereas columns were ordered according to forests.

The core community was inferred and visualized by first transforming raw count numbers into community proportions, and then selecting those ASVs that were present in all samples. Then, ASV occurrences in forest types were plotted in a Venn diagram using the package VennDiagram (Chen and Boutros, 2011). Further, ASV sequences of the core community were blasted against NCBI type material to find their closest culturable relatives and thus to retrieve potential metabolic capabilities. Finally, ASV sequences were searched within the complete NCBI database and the location of the top five blast entries (*P*_ident_ > 0.95) was retrieved to have an idea of common habitats for the core community members.

### Biogeographic analysis

We rarefied samples to equal sequencing depth (9,843 counts). Afterward, prokaryotic community overlap was measured using the mean Jaccard overlap (MJO) which ranges from 0 (no ASV overlap between pair of samples) to 1 (complete ASV overlap). Then, we compared the measured MJO against a null model. The null model was designed by randomly assigning counts to ASVs, proportional to their mean relative abundance based on a multinomial distribution, while preserving the total number of ASVs per sample and regional pool (Louca et al., 2017b). The significance of the observed MJO was derived from the fraction of MJOs calculated for 1,000 shuffled datasets that were lower or equal the observed value. To quantify the variability of ASV abundances within the dataset, we calculated the coefficient of variation (CV) as the standard deviation divided by the mean of ASV relative abundances. We calculated CVs for each vegetation type individually by averaging over all occurring ASVs within the respective sample group, as well as for the global dataset. Moreover, we applied a generalized Morisita similarity index (MA) according to Chao et al. (2008) and compared the observed MA score to the MA score distribution retrieved from shuffling the dataset 1,000 times using a null model approach which assigns individual counts to each matrix cell proportional to the total row and column sums until total abundance is reached (Ulrich and Gotelli, 2010; Louca et al., 2017b). A significant lower MA score as compared to the null model indicates segregation of ASVs between samples, whereas a significant higher MA score indicates aggregation.

## Results

### Taxonomy

**Figure 1:**
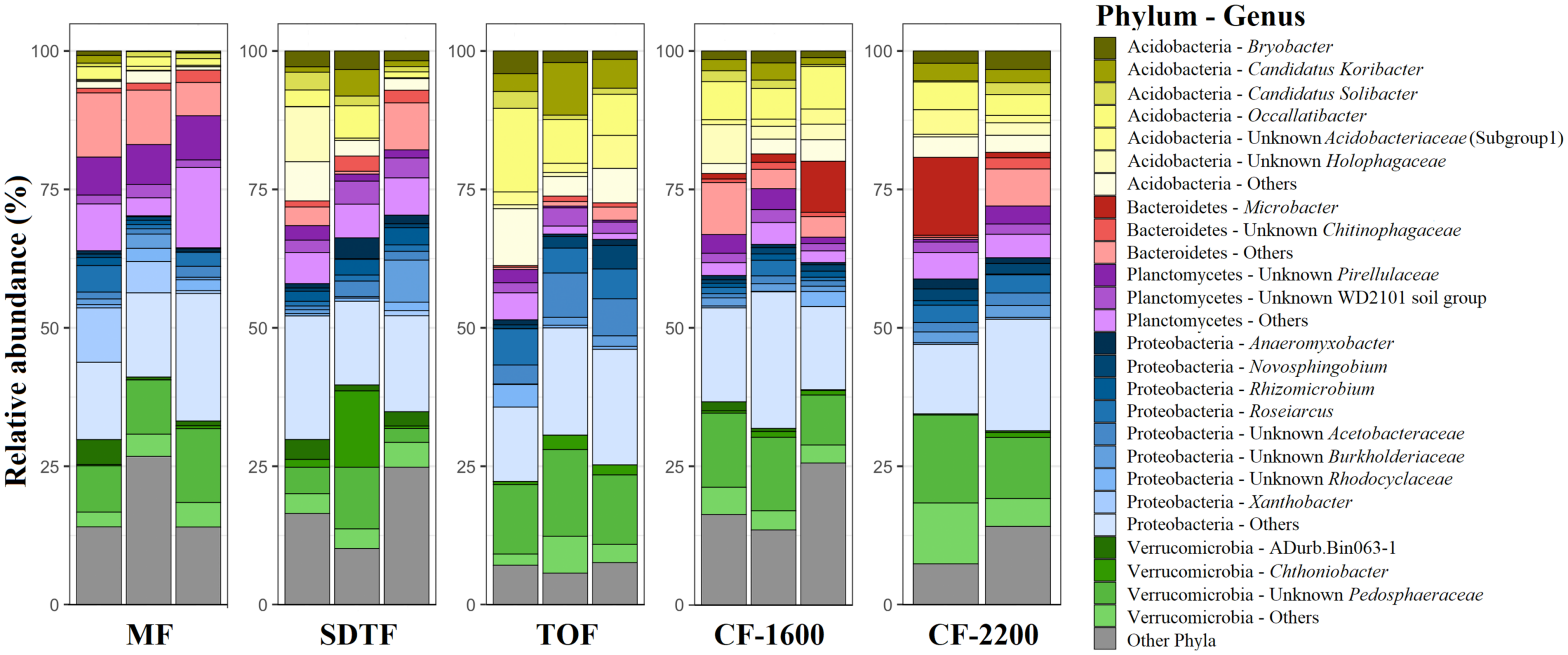
Relative abundance of prokaryotes (archaea and bacteria) phyla and genera in tank bromeliads in five different forests along an elevation gradient in Veracruz, Mexico. Each bar represents a sample (bromeliad). Phyla and genera contributing < 1% to the complete dataset were grouped into “others.” Mangrove forest (MF), semi-deciduous tropical forest (SDTF), tropical oak forest (TOF), and cloud forest (CF).

**Figure 2:**
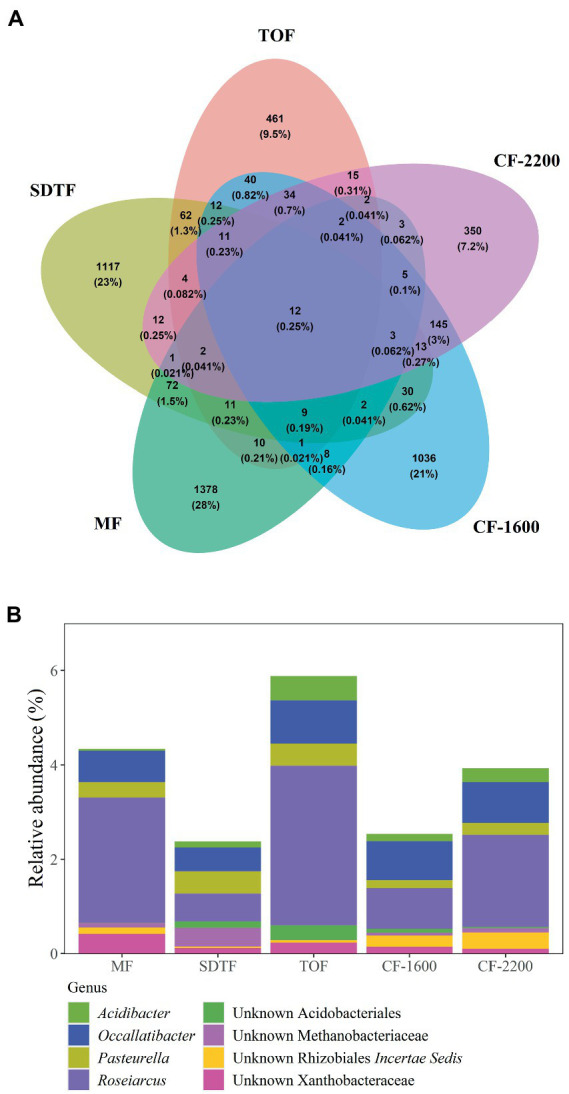
Venn diagram showing shared and unique ASVs between five different forests **(A)**, and relative abundance of ASVs found in all bromeliads in all forests, core community **(B)**. Mangrove forest (MF), semi-deciduous tropical forest (SDTF), tropical oak forest (TOF), and cloud forest (CF).

In total we generated 1,436,874 reads of which we recovered 273,772 after bioinformatic processing with a mean sequencing depth of 19,555 reads per sample. Most sequences were discarded during quality filtering. With 4,863 ASVs we found a remarkably high prokaryotic diversity in tank bromeliads along the elevation gradient. The bacterial community consisted of 38 phyla and 85 classes or equivalents (candidate taxa), and the archaeal community consisted of three phyla and five classes (Supplementary Figure S1). Proteobacteria represented the most abundant phylum in all study sites with *ca*. 30% of the identified ASVs, followed by Verrucomicrobia (18%), Acidobacteria (17%), Planctomycetes (12%), and Bacteroidetes (8%; Figure 1). At the genus level, the dominant taxa in all forests (>5% of the reads of each site) were unidentified members of the family Pedosphaeraceae, in addition to Pirellulaceae and *Xanthobacter* in the mangrove (MF), *Chthoniobacter* in the semi-deciduous tropical forest (SDTF), *Occallatibacter* in the tropical oak forest (TOF), and cloud forest 1,600 m a.s.l. (*CF*-1600), and *Microbacter* in the *CF*-2200 (Figure 1). Methanobacteria and Methanomicrobia were by far the most abundant archaea representing *ca*. 26 and 60%, respectively, of all the identified organisms of this domain (Supplementary Figure S1). The core community accounted for 2–6% of the reads in the studied forests but represented only 0.25% of the total of identified taxa (Figures 2A,B). It was composed of 12 ASVs belonging to seven bacterial taxa, as well as one archaeal taxon (Figure 2B). Most of the closest related described species of this core community were commonly found in acidic or disturbed soils, sediments, and sewage (Table 1).

**Table 1:** Closest related type species of the bacterial core community ASVs (sorted according to their class affiliation) identified after blast analysis (www.ncbi.nlm.nih.gov/blast).

Class	ASV classification	Closest type species (Acc. no)	Sim (%)	Location of the five closest related type species/sequences (Acc. no)
Acidobacteriia	Unknown Acidobacteriales	*Acidobacterium ailaaui* (NR_153719.1)	92.8	Rice straw anaerobic digester (MG854131.1); subsurface paddy soil, China (MG100884.1); acidic fen soil, Germany (GU127783.1); wetland sediments, China (KX823763.1); rice rhizosphere, Indonesia (KT199267.1)
	Unknown *Occallatibacter*	*Occallatibacter savannae* (NR_147737.1)	98.0	Tobacco rhizosphere in soil with fungicide (KM200481.1); terrestrial ecosystems, United States (JQ382427.2); turf grass field, United States (JQ358450.1); long-term agroecological research site, United States (HM062092.1); soil communities in agriculture, United States (EF665275.1)
	Unknown *Occallatibacter*	*Terracidiphilus gabretensis* (NR_146368.1)	96.8	Soil, United States (FJ166066.1); bog soil, Germany (LN715646.1); forest soil, Taiwan (JN851472.1); oil palm rhizosphere, Malaysia (JX522849.1); wood land soil, Germany (HQ598923.1)
Alpha-proteobacteria	Unknown Rhizobiales Incertae Sedis	*Aestuariivirga litoralis* (MH371374.1)	94.8	Forest ecosystem, New-Zealand (MH531180.1); rhizosphere of *Lythrum anceps*, Japan (LC378722.1); mangrove soil sample, Brazil (JN817765.1); Lake Naheul Huapi, Argentina (KM135805.1); rhizoplane of Japanese loosestrife (AB529696.1)
	Unknown *Roseiarcus*	*Roseiarcus fermentans* (NR_134158.1)	99.5	Peat swamp forest soil, Thailand (GQ402766.1); disturbed field soil, China (EU881257.1); floodplain lake water, Brazil (MF439799.1); anaerobic UASB reactor with sewage, Japan (LC256314.1); bog soil, Germany (LN715564.1)
	Unknown *Roseiarcus*	*R*. *fermentans*	96.3	Rice soil, China (KJ587537.1); soil sample, USA (JQ387066.2); root of aquatic plant, Japan (LC106260.1); soil from a natural CO_2_ spring, Slovenia (HF952340.1); methanotrophic plant-symbiont, Netherlands (AY163571.1)
	Unknown *Roseiarcus*	*R*. *fermentans*	97.2	Fen soil, Germany (LN716042.1); bog soil, Germany (LN715527.1); forest soils, China (JX885273.1); groundwater discharge zone sediment, Canada (KC922689.1); forest soil, France (HQ629067.1)
	Unknown *Roseiarcus*	*R*. *fermentans*	98.5	Water sample, Taiwan (MG993536.1); fen soil, Germany (LN716042.1); forest soil, China (JX885273.1); *Kobresia* meadow soil, Tibetan Plateau, China (GQ127783.1); forest soil, New Zealand (MH530691.1)
	Unknown Xanthobacteraceae	*Pseudolabrys taiwanensis* (NR_043515.1)	96.5	Rice straw anaerobic digester, United Kingdom (MG852539.1); anaerobic UASB reactor with sewage, Japan (LC246547.1); forest soil, New Zealand (MH531876.1); sewage sludge, China (MG803803.1); contaminated meadow soil, Bulgaria (MW899593.1)
Gamma-proteobacteria	Unknown Acidibacter	*Acidibacter ferrireducens* (NR_126260.1)	94.4	Forest soil, New Zealand (MH524718.1); lake water, Canada (KY519894.1); forest and grassland soil, Taiwan (EU849406.1); volcanic ash deposit, Japan (AB552254.1); granite outcrop plant rhizosphere, USA (KY992630.1)
	Unknown *Pasteurella*	*Pasteurella multocida* (NR_115136.1)	100	Cat oral cavity, Iraq (MN588319.1); swine, China (MK234576.1); musk deer, China (MN080875.1); goat blood, India (MH068782.1); sheep lung tissue, India (MF417604.1)

The similarity (Sim) shows the match in bp between ASVs and type species. The locations of the top five entries were taken from sequences with >95% of similarity against bacterial core community ASVs, repeated entries were omitted. Accession numbers (Acc. No) are given in parentheses.

### Alpha and beta diversity

We calculated effective number of species (ENS) to compare alpha-diversity between forests. Bromeliads in the SDTF showed the highest ENS (114), closely followed by the *CF*-2200 (104) and the *CF*-1600 (101). The lowest ENS was found in the MF (73) and the TOF (70). However, differences in ENS between forests were not significant [H_(4)_ = 2.1, *p* = 0.72]. Most of the ASVs in tank bromeliads (88.7%) were found only in one forest type (average proportion of the relative abundances of all shared ASVs 3.81% ± 1.97 SD), being 28% exclusive to the MF, 23% to the SDTF and 21.3, 9.5 and 7.2% to the *CF*-1600, *CF*-2200 and TOF, respectively (Figure 2A). A high proportion of ASVs was not shared between samples within forests either: only 4% of the ASVs in the MF were found in all MF samples, 2% in the SDTF, 11% in the TOF, 6% in the *CF*-1600 and 13% in the *CF*-2200 (Supplementary Figure S2). Despite the dominance of forest specific ASVs, the taxonomic composition of each forest community at higher taxonomic ranks was quite similar, indicating similar environmental conditions within forests (Figure 1). When we grouped ASVs according to their abundance distribution within the dataset, we found that the distribution of ASVs was sorted into forest specific clusters, irrespective of the sampled bromeliad species. This pattern was consistent among different taxonomic groups (Supplementary Figure S3).

### Environment, biogeography and prokaryotic community composition

**Figure 3:**
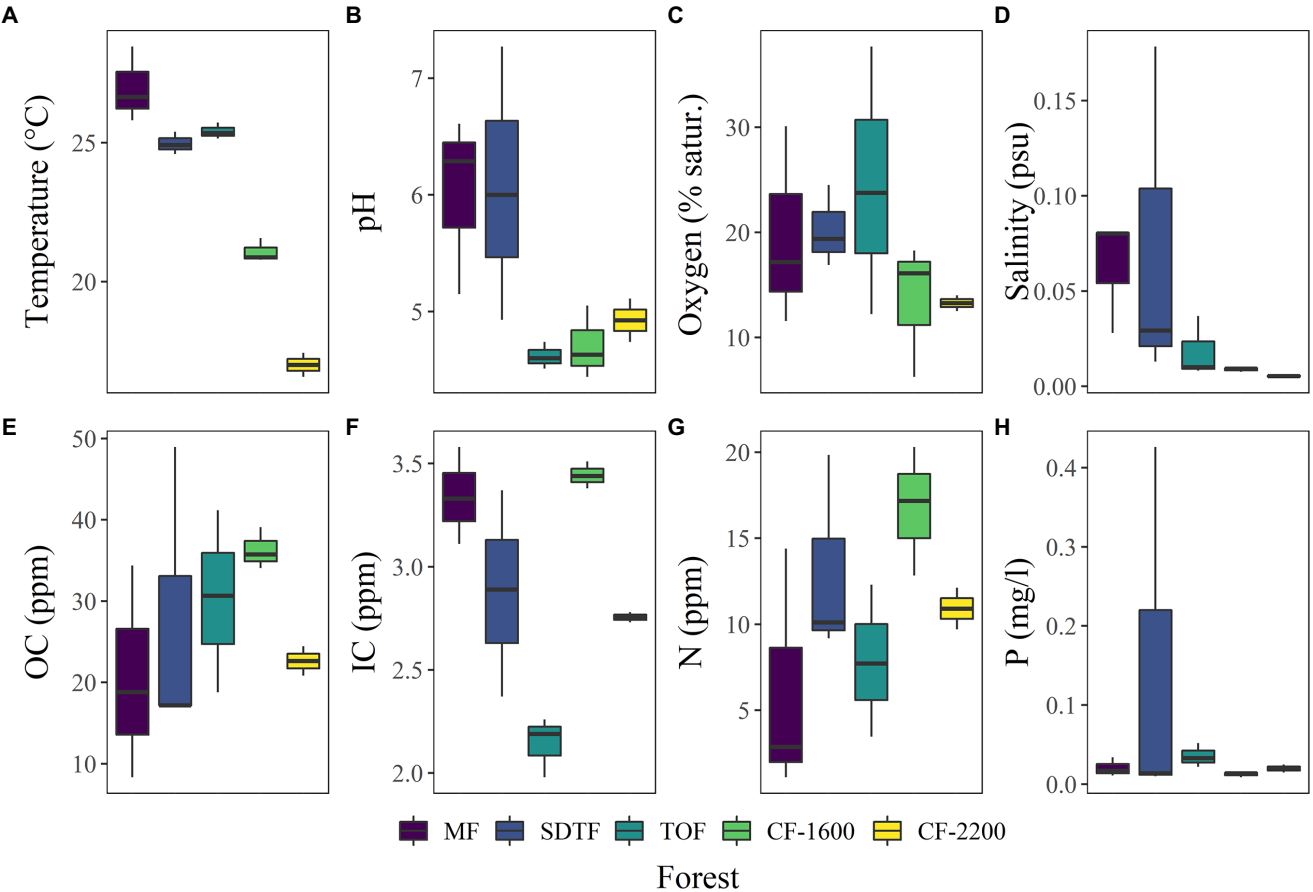
**(A–H)** Box plots of physicochemical characteristics of bromeliad water, measured in 14 plants in five different forests along an elevation gradient in Veracruz, Mexico. Mangrove forest (MF), semi-deciduous tropical forest (SDTF), tropical oak forest (TOF), and cloud forest (CF).

Bromeliad tank water was acidic (pH = 5.3 ± 0.89, mean ± SD, *n* = 14, Figure 3B), low in dissolved oxygen (<40% saturation, Figure 3C), fresh (salinity <0.2 PSU, Figure 3D), and rich in carbon (OC = 27.8 ± 11.5 ppm, IC = 2.9 ± 0.5 ppm, Figures 3E,F) and nutrients (*N* = 10.9 ± 5.9 ppm, *P* = 0.05 ± 0.1 mg/l, Figures 3G,H). According to the PCA performed using these physicochemical parameters, tank water of bromeliads from the same forest tended to cluster. However, of all the measured parameters only temperature [H_(4)_ = 12.0, *p* = 0.017], salinity [H_(4)_ = 9.7, *p* = 0.045] and IC [H_(4)_ = 10.5, *p* = 0.03] were significantly different between forests, while pH was not significant, although marginally [H_(4)_ = 9.2, *p* = 0.057].

**Figure 4:**
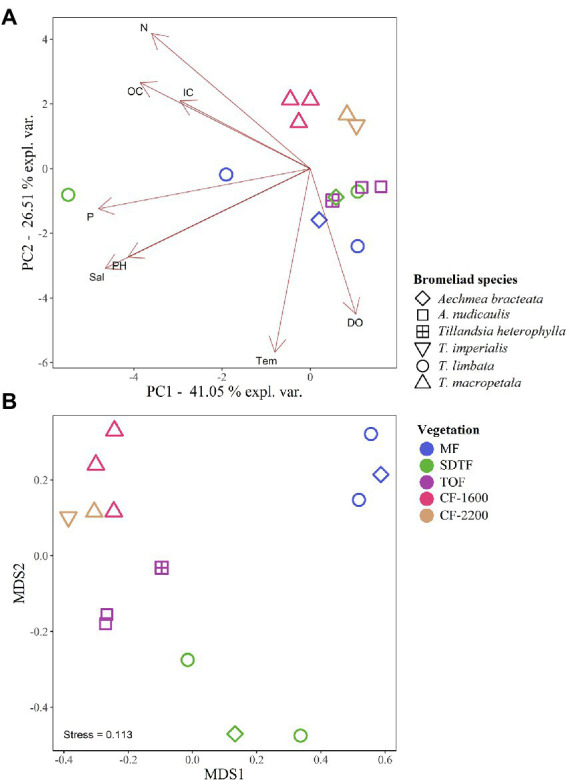
Principal component analysis (PCA) of physicochemical characteristics of bromeliad water **(A)**, and non-metric multidimensional scaling (NMDS) of the prokaryotic community composition **(B)**, in five different forests along an elevation gradient in Veracruz, Mexico. Mangrove forest (MF), semi-deciduous tropical forest (SDTF), tropical oak forest (TOF), and cloud forest (CF). Dissolved oxygen (DO), inorganic carbon (IC), nitrogen (N), organic carbon (OC), phosphorus (P), salinity (Sal), and temperature (Temp).

The PERMANOVA of Bray-Curtis dissimilarities suggests an effect of forest type (*R*^2^ = 0.44, *p* = 0.014) on the prokaryotic composition in the tanks of bromeliads, and the NMDS supports this notion: prokaryotic communities in bromeliads were more similar within than between forests (Figure 4B). We also detected three abiotic parameters that significantly covaried with prokaryotic community changes: temperature (*R*^2^ = 0.122, *p* = 0.036), pH (*R*^2^ = 0.113, *p* = 0.014) and IC (*R*^2^ = 0.111, *p* = 0.036). All three parameters together explained 32% of compositional community variation.

According to the high compositional variance among samples within and between forests, we found that the Mean Jaccard Overlaps (MJO) in all forests ranged from 0.01 to 0.25 (global MJO = 0.05). These values were significantly lower than expected from random sampling of the regional organism pool (*p* < 0.001). Moreover, the coefficient of variation indicated a higher variability of the abundances of each ASV between forests (CV = 3.5) than within forests (CVs 1.3–1.7), and according to the generalized Morisita similarity index (MA) all samples expressed segregation effects that were significantly stronger than inferred from null models (*p* < 0.001).

### Functional diversity

**Figure 5:**
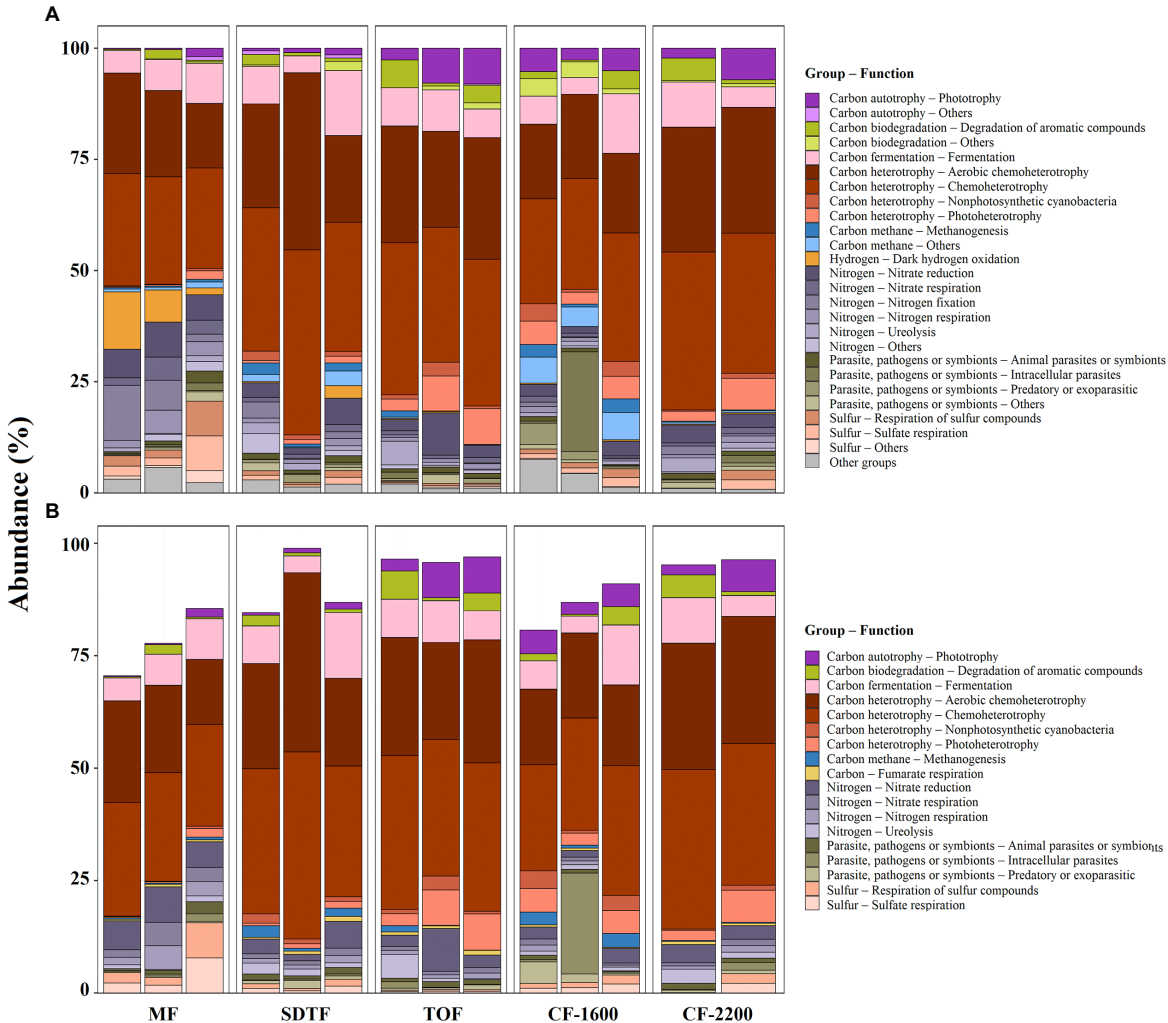
Relative abundance of ASVs associated with putative metabolic groups **(A)**, and relative abundance of ASVs associated with putative metabolic groups present in all samples, core metabolic groups **(B)**, in five different forests along an elevation gradient in Veracruz, Mexico. Each bar represents a sample (bromeliad). Mangrove forest (MF), semi-deciduous tropical forest (SDTF), tropical oak forest (TOF), and cloud forest (CF).

The functional annotation of ASVs revealed a high richness of putative metabolic functions within prokaryotic communities in tank bromeliads, represented by 61 functional groups according to FAPROTAX. The functional community was dominated by chemoheterotrophs (>25% of assignments), fermenters (*ca*. 8%), nitrate reductors (*ca*. 5%), and nitrogen fixers (4%; Figure 5A). Hydrogen-oxidizing, photoheterotrophic, sulfate-respiring, and intracellular parasitic prokaryotes were also abundant, and all together comprised *ca*. 10% of the assigned organisms. About 70% of the inferred functional groups contributed less than 1% to the total assignments (Figure 5A).

In contrast to the small proportion of ASVs shared within and between forests in tank bromeliads, there was high redundancy of putative metabolic functions in the tanks of these plants, with *ca*. 30% of the identified metabolic groups being present in all samples (core metabolic group, Figure 5B). Although the core metabolic group was diverse, almost 80% of all ASVs that could be functionally annotated were chemoheterotrophs, fermenters and nitrate reductors (Figures 5A,B). The contribution of functional groups with lower abundances within the core metabolic group was highly variable between forests, e.g., more than 50% of the putative methanogenic archaea were found in the SDTF and *CF*-1600, and *ca*. 80% of the putative sulfate reducers in the MF (Figure 5B).

## Discussion

### Prokaryotic communities

This study revealed rich prokaryotic communities in tank bromeliads along an elevation gradient in tropical Mexico, confirming these plants as extremely diverse micro-habitats for bacteria and archaea in the Neotropics (Goffredi et al., 2015; Louca et al., 2017b; Rodriguez-Nuñez et al., 2018; Giongo et al., 2019; Simão et al., 2020). Consistent with previous studies based on amplicon sequencing of 16S rRNA genes (Goffredi et al., 2011; Louca et al., 2017b; Rodriguez-Nuñez et al., 2018; Simão et al., 2020), Proteobacteria, Verrucomicrobia, Planctomycetes, Acidobacteria and Bacteroidetes were among the top 10 most abundant prokaryotic groups in these plants, comprising 85% of the ASVs in our samples. Interestingly, a similar composition was found in soils, where *ca*. 83% of the libraries were comprised of members of these phyla (Janssen, 2006). However, in comparison to average values for soil (Janssen, 2006) the relative abundance of Planctomycetes and Verrucomicrobia in tank bromeliads was higher. This was not unexpected considering that Planctomycetes have been commonly recovered from water-saturated environments, rich in plant-derived organic matter and with a low pH, such as peat bogs and other acidic bromeliad tanks (Kulichevskaya et al., 2007; Moore et al., 2015; Dedysh and Ivanova, 2019). Moreover, Verrucomicrobia are abundant in heavily polluted or eutrophic aquatic habitats including bromeliad phytotelmata (Schlesner et al., 2006; Bergmann et al., 2011; Louca et al., 2017a). In contrast to these two phyla, the proportion of Actinobacteria, one of the most abundant bacteria groups in soils (Lewin et al., 2016; Delgado-Baquerizo et al., 2018) was relatively low in our samples (<10% of the ASVs), probably due to the acidic conditions that prevailed in most tanks. Thus, in general, Actinobacteria are positively correlated with pH (Lauber et al., 2009).

The blast analysis of the core community assigned most of the ASVs to organisms that occur in forest and agricultural soils, some of which were attributed to the rhizosphere of several plants. Moreover, four ASVs were attributed to *Roseiarcus fermentans*, a bacteriochlorophyll-a containing fermentative bacterium, typically found in wetlands and peat-soil (Kulichevskaya et al., 2014). Searches within the complete NCBI database revealed that most representative type-material was culturable at low pH (≥3) and was able to degrade plant-derived biopolymers and organic matter, indicating a prevalence toward acidic environments rich in organic carbon.

The core community also included one ASV belonging to *Pasteurella multocida*, an opportunistic pathogen that can cause avian cholera and is highly abundant in the oral or nasopharyngeal microbiota of animals (Botzler, 1991; Davies, 2004). This is an important discovery considering that *P*. *multocida* infects over 100 wild avian species and annually kills thousands of waterfowl in North America, but their reservoirs (places where the infective agent can survive on a year-round basis) are still largely unknown (Botzler, 1991; Samuel et al., 2004). Although wetlands have long been suspected to be an important reservoir for this pathogen (Samuel et al., 2004), previous studies showed that *P*. *multocida* can survive only for short periods in water and sediments following cholera outbreaks (Samuel et al., 2004; Blanchong et al., 2006). Interestingly, we found *P*. *multocida* in all our samples, even though they were collected in very different forests. However, we only sequenced short reads (444 bp) of the V3-V4 region of the 16S rRNA gene. This hampers the identification of *P*. *multocida* strains and capsular serogroups, which is necessary to associate *P*. *multocida* with specific diseases including fowl cholera (Davies, 2004). Future investigations using longer reads of the 16S rRNA gene, including periodic sampling in tank bromeliads over a one-year period, especially in bird migratory routes, are still needed to identify the strain of this possible pathogen and to understand if bromeliads may play a role in avian cholera.

### Environment and biogeography

While the prokaryotic diversity along the elevation gradient was remarkable, differences in alpha-diversity were not detected among forests. This result indicates that, similarly to soil bacteria across biomes (Walters and Martiny, 2020), tank bromeliads are suitable habitats that contain a high diversity of prokaryotes within a single sample regardless of forest type. In general, tank bromeliads provide nutrient-rich and wet habitats (Richardson et al., 2000; Zotz et al., 2020; Aguilar-Cruz et al., 2022), where environmental factors can greatly differ depending on the time, plant structure, and position in the canopy (Laessle, 1961; Haubrich et al., 2009; Brouard et al., 2012; Brandt et al., 2017; Giongo et al., 2019). Therefore, these tanks offer a large environmental heterogeneity even within forests and promote prokaryotic diversity at a local scale.

The low ASV overlap between forests suggests a deviation from neutral expectations, which may indicate that deterministic processes are mainly responsible for shaping the prokaryotic composition along the elevation gradient (Livermore and Jones, 2015). However, in this study, we cannot formally test the effect of dispersal limitation due to the limited data set and the lack of a sequential sampling along the elevation gradient. Nonetheless, the low importance of dispersal effects on the local community assembly in tank bromelias (Louca et al., 2017b), in addition to the low MJOs observed between our samples suggest that environmental selection has a higher impact on prokaryotic communities. Under a non-neutral assumption, the covariation of forest type with prokaryotic composition could indicate selection mechanisms that promote similar compositions under similar environmental conditions within forests. This interpretation is also supported by the generalized Morisita index analysis, which indicated segregation effects that were significantly stronger than inferred from the null models in all samples, pointing toward environmental filtering or negative biotic interactions between organisms. However, from all the measured environmental parameters only temperature, pH, and inorganic carbon significantly covaried with prokaryotic community differences, and together explained just one-third of the compositional community variation, leaving large potential for various assembly processes.

The low predictive power of environmental variables is a common observation in prokaryotic studies in tank bromeliads and suggests that additional unstudied factors, such as biotic interactions, immigration or environmental disturbances, are also important at the community level (Farjalla et al., 2012; Louca et al., 2017b). Vegetal litter constantly enters the tanks (Aguilar-Cruz et al., 2022) and animals are often in contact with tank water and litter (Nadkarni and Matelson, 1989; Thorne et al., 1996; Aguilar-Cruz et al., 2021). This was corroborated by the presence of putative animal parasite or symbionts in all samples (Figure 5B). Each of these events allows prokaryotic immigration of new organisms from outside of the regional organism pool, and even though it has been experimentally shown that early prokaryotic colonizers are replaced by organisms better adapted to the environmental conditions (Brislawn et al., 2019), regular migration can sustainably alter prokaryotic community composition (Dottorini et al., 2021). Moreover, strong environmental disturbances such as a drought, have the potential to greatly change the prokaryotic communities (Brandt et al., 2015; Meisner et al., 2018). Consequently, the individual history of bromeliads is likely to play a major role for their tank associated prokaryotic community composition and explain the relatively high compositional variation between tank communities and the low predictive power of environmental variables.

Nonetheless, our results suggest that pH is an important driving factor for prokaryotic composition in these microecosystems. This supports previous findings showing that variation in bacterial communities were correlated with differences in pH in bromeliad water (Goffredi et al., 2011). Our results also showed that prokaryotic community variation significantly covaried with inorganic carbon, but not with organic carbon, probably due to the high OC/IC ratio that prevailed in the tanks. Thus, although even small changes in OC can influence the prokaryotic community composition in oligotrophic systems, community composition is more resistant to change under eutrophic conditions (Eiler et al., 2003). Interestingly, we also found that temperature affected prokaryotic community composition, even though previous studies performed along elevation gradients suggested that this environmental variable may have a weak or no effect on prokaryotic alpha and beta diversity in terrestrial ecosystems (Fierer et al., 2011; Lanzen et al., 2016; Peay et al., 2017). However, our results should be interpreted with caution because IC and temperature in tank bromeliads were significantly different between forests, and with our data it is not possible to distinguish if the observed compositional differences were directly linked to these variables or to other parameters that acted as confounding factors at a local scale. Moreover, temperature varies during the day in bromeliad phytotelmata (Laessle, 1961; Louca et al., 2017a) and we only measured it for a short time period. Nevertheless, daily temperature variation may have a negligible impact on prokaryotic community composition considering that along elevation gradients spatial patterns may be the result of long-term rather than short-term site-specific temperatures regimes (Frindte et al., 2019).

Furthermore, we cannot disentangle the effect of vegetation type from bromeliad species on microbial composition, because not all bromeliads were found in all forests. Even though some prokaryotic groups may be species-specific (Vergne et al., 2021), our heatmaps of taxonomic groups found along the elevation gradient revealed a distinct clustering of ASVs according to forest type but not bromeliad species. This result suggests that the effect of bromeliad species on prokaryotic composition is minor compared to vegetation type and may be related to changes in the tank environment and not the species *per se* (Louca et al., 2017a). For example, Carrias et al. (2014) found that algal communities of two coexisting tank bromeliads, growing in proximity, cluster according to bromeliad species. However, the differences in community structure were linked to differences in the aquatic habitat, which is directly related to the species of tank-forming plant. In addition, Goffredi et al. (2011) performed a cluster analysis of bacterial communities of three bromeliad species and found that pH has a stronger influence on bacterial community composition than bromeliad species.

### Putative metabolic functions

We found a high diversity of putative metabolic functions in tank bromeliads along the elevation gradient. However, we stress that in this work we did not directly study metabolic processes, and the metabolic functions were only inferred from 16S rRNA sequences using FAPROTAX. Many organisms known to perform certain functions may be missing in FAPROTAX, a taxon may be affiliated with multiple functions and functional groups may be nested, for example denitrifying taxa are also associated with nitrate respiration.^1^ Moreover, we sampled tank bromeliads that were located on the trunk or inner branches of trees, characterized by a high input of allochthonous organic matter, which is derived mainly from tree litter (Aguilar-Cruz et al., 2022). This was reflected not only in the environmental conditions inside the tanks, with high concentrations of OC, low pH and O_2_, but also in the relative abundance of the putative metabolic functions of prokaryotes. Thus, taxonomic groups involved in the biological cycling of OC, including chemoheterotrophs and fermenters, dominated the prokaryotic communities along the elevation gradient. Similarly, a study focused on invertebrates showed that over a broad geographic range, aquatic food webs of tank bromeliads are mostly allochthonous-based (Farjalla et al., 2016), and chemoheterotrophic prokaryotes are consistently dominant in these plants in diverse ecosystems (Goffredi et al., 2011; Louca et al., 2017a; Herrera-García et al., 2022). These findings support the hypothesis that bromeliads act as natural biodigesters in Neotropical forests and potentiate the decomposition process aboveground (Aguilar-Cruz et al., 2020). In fact, our results show that this analogy is quite appropriate considering that some of the most abundant chemoheterotrophic groups found in these plants, such as Xanthobacter and Sphingomonadaceae, are able to degrade organic substances, including alcohols, organic acids, and aromatic compounds, and have been used in the activated sludge of wastewater treatment plants (Glaeser and Kämpfer, 2014; Oren, 2014a). In addition, in all samples we found archaeal species belonging to Methanobacteriaceae that use H_2_ and CO_2_ as a substrate for methanogenesis and are abundant in anaerobic digestors (Oren, 2014b). Their presence within the dataset was surprising as the applied V3-V4 primer set is known to discriminate against archaeal sequences (Wear et al., 2018), hence underlining their importance for the studied system. These archaea seem to be omnipresent in tank bromeliads, where they are well-adapted to the organic carbon rich and oxygen-limited environment at the bottom of the tanks (Louca et al., 2017a). In association with heterotrophic bacteria, Methanobacteriaceae may contribute substantially to the anaerobic degradation of carbon and the production of CH_4_ (Martinson et al., 2010).

Although we collected samples from bromeliads that were not exposed to full sunlight, we found phototrophic bacteria in all our samples. Most of them were purple non-sulfur bacteria (PNSB) including Azospirillales, Rhodobacterales, and Rhodospirillales belonging to α-proteobacteria, as well as Rhodocyclaceae belonging to β-proteobacteria. These organisms are metabolic versatile and may be abundant in the interface between aerobic and anaerobic zones in the tanks of these plants. Thus, many species grow organoheterotrophically in aerobic or microaerophilic environments, and under anoxic conditions by either fermentation or anaerobic respiration in the dark, or chemolithoautotrophically with H_2_ or low levels of sulfide as photosynthetic electron donors (Madigan and Jung, 2009). Although purple sulfur bacteria (PSB) belonging to Ectothiorhodospirales were also found, they were much less abundant than PNSB, probably because sulfate is severely depleted in the tanks of bromeliads, and the physiology of PSB is intimately linked to sulfide (Madigan and Jung, 2009; Louca et al., 2017a).

Our results also indicate that prokaryotes actively participate in the nitrogen cycle in tank bromeliads. Taxonomic groups related to the hydrolysis of urea to NH_4_^+^, one of the most important nitrogen forms for the nutrition of these plants (Inselsbacher et al., 2007), were present in more than 50% of the samples. Furthermore, except for one plant in the TOF, we found putative nitrogen fixers in all bromeliads along the elevation gradient, supporting previous findings of genes associated with nitrogen fixation in these plants (Goffredi et al., 2011; Louca et al., 2017a). However, from all detected groups involved in the nitrogen cycle, nitrate reductors were ubiquitous and the most abundant. These results support the notion that in these microecosystems more nitrogen is lost by dissimilatory reduction as N_2_ than what is fixed by prokaryotes, and that possibly nitrogen inputs counteract denitrification and provide sufficient nitrogen for assimilation (Louca et al., 2017a).

## Conclusion

This study shows that even in very different forests, tank bromeliads hosted a high diversity of prokaryotes, largely exclusive to only one forest type. Therefore, these plants represent key secondary foundation species (Thomsen et al., 2018), which promote prokaryotic richness at a local and regional scale in the Neotropical forest canopies. These prokaryotic communities are probably shaped by non-neutral processes such as environmental filtering, and they significantly covaried with environmental factors inside the tanks. These environmental factors tend to be more similar in bromeliads within forests, which may promote the presence of more similar prokaryotic communities within than between vegetation types. However, organisms commonly found in acidic environments, rich in organic matter and low in dissolved oxygen were present in all bromeliads along the elevation gradient, showing that despite the low compositional overlap there are common niches offered by these plants that are occupied by similar taxa. Furthermore, in accordance with a previous study (Louca et al., 2017b) we found a high redundancy of putative metabolic functions in tank bromeliads. This suggests that even across environmental gradients there are metabolic functions that are relatively constant in these microecosystems, most of them related to the biological cycling of organic carbon and nitrogen.

## Data availability statement

The datasets presented in this study can be found in online repositories. The names of the repository/repositories and accession number(s) can be found at: https://www.ebi.ac.uk/ena, PRJEB52870.

## Author contributions

YA-C, GZ, and TB conceived and designed the study. YA-C, JL, and AP carried out the laboratory work. FM and YA-C performed the bioinformatics and statistical analysis. FM carried out the biogeographic analysis. All authors contributed to the article and approved the submitted version.

## Funding

YA-C was funded by the Consejo Nacional de Ciencia y Tecnología (CONACyT 408928), Deutscher Akademischer Austauschdienst (DAAD 91569882), and Heinz Neumüller Stiftung.

## Conflict of interest

The authors declare that the research was conducted in the absence of any commercial or financial relationships that could be construed as a potential conflict of interest.

## Publisher’s note

All claims expressed in this article are solely those of the authors and do not necessarily represent those of their affiliated organizations, or those of the publisher, the editors and the reviewers. Any product that may be evaluated in this article, or claim that may be made by its manufacturer, is not guaranteed or endorsed by the publisher.

## Footnotes

^1^ www.zoology.ubc.ca/louca/FAPROTAX
