# Supplementary material for: Diversity and putative metabolic function of prokaryotic communities in tank bromeliads along an elevation gradient in tropical Mexico
Source: Front Microbiol. 2022 Oct 13;13:945488. doi: 10.3389/fmicb.2022.945488 (PMC9608151; doi:10.3389/fmicb.2022.945488)
Supplement: Supplementary file 1 [file Data_Sheet_1.pdf]

frontiers

Diversity and putative metabolic function of prokaryotic communities in tank bromeliads along an elevation gradient in tropical Mexico

Yonatan Aguilar-Cruz, Felix Milke, Janina Leinberger, Anja Poehlein, Gerhard Zotz, Thorsten Brinkhoff.

Supplementary material

Table S1. Settings used in polymerase chain reaction (PCR).

Reagent	Volume [µl]	Cycles	Step	Temperature [°C]	Time [min]
5x Phusion GC buffer*	10	1	Initial denaturation	98	01:00
DEPC H_2O	30.8				
Primer V3f (20 pmol)	1	30	Denaturation	98	00:45
Primer V4r(20 pmol)	1		Primer annealing	60	00:45
$MgCl_2$*	0.2		Elongation	72	00:30
DMSO*	2.5				
BSA (10 mg/ml)	1	1	Final elongation	72	05:00
dNTPs (10 mM)	1				
Phusion Polymerase	0.5				
Template	2				
Sum	50		Hold	4	until end

*supplied with Phusion High Fidelity DNA Polymerase (New England BioLabs)

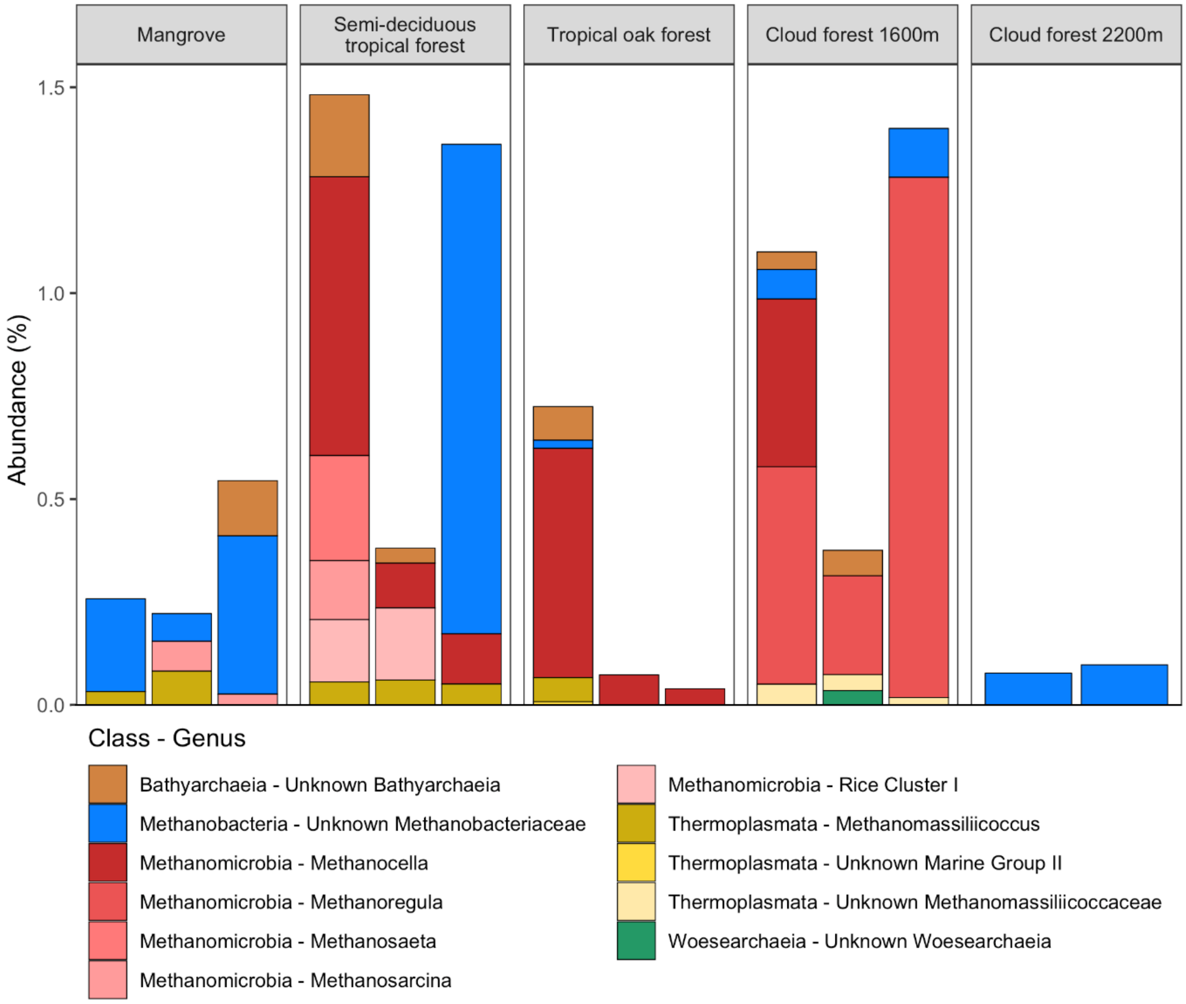

Figure S1. Relative abundance of archaea classes and genera in tank bromeliads in five different forests along an elevation gradient in Veracruz, Mexico. Each bar represents a sample (bromeliad).

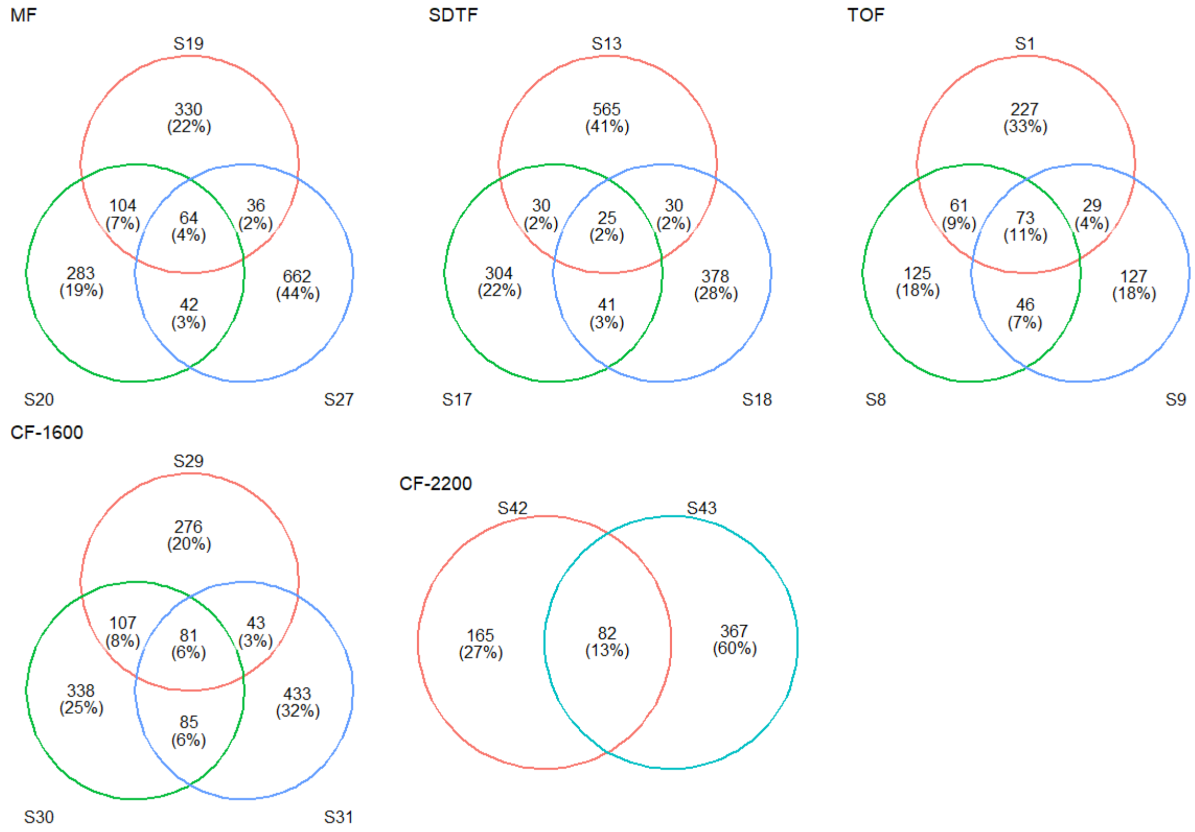

Figure S2. Venn diagrams showing the distribution of ASVs within forests. Mangrove forest (MF), semi-deciduous tropical forest (SDTF), tropical oak forest (TOF) and cloud forest (CF). Sample IDs are represented by the letter S followed by a number.

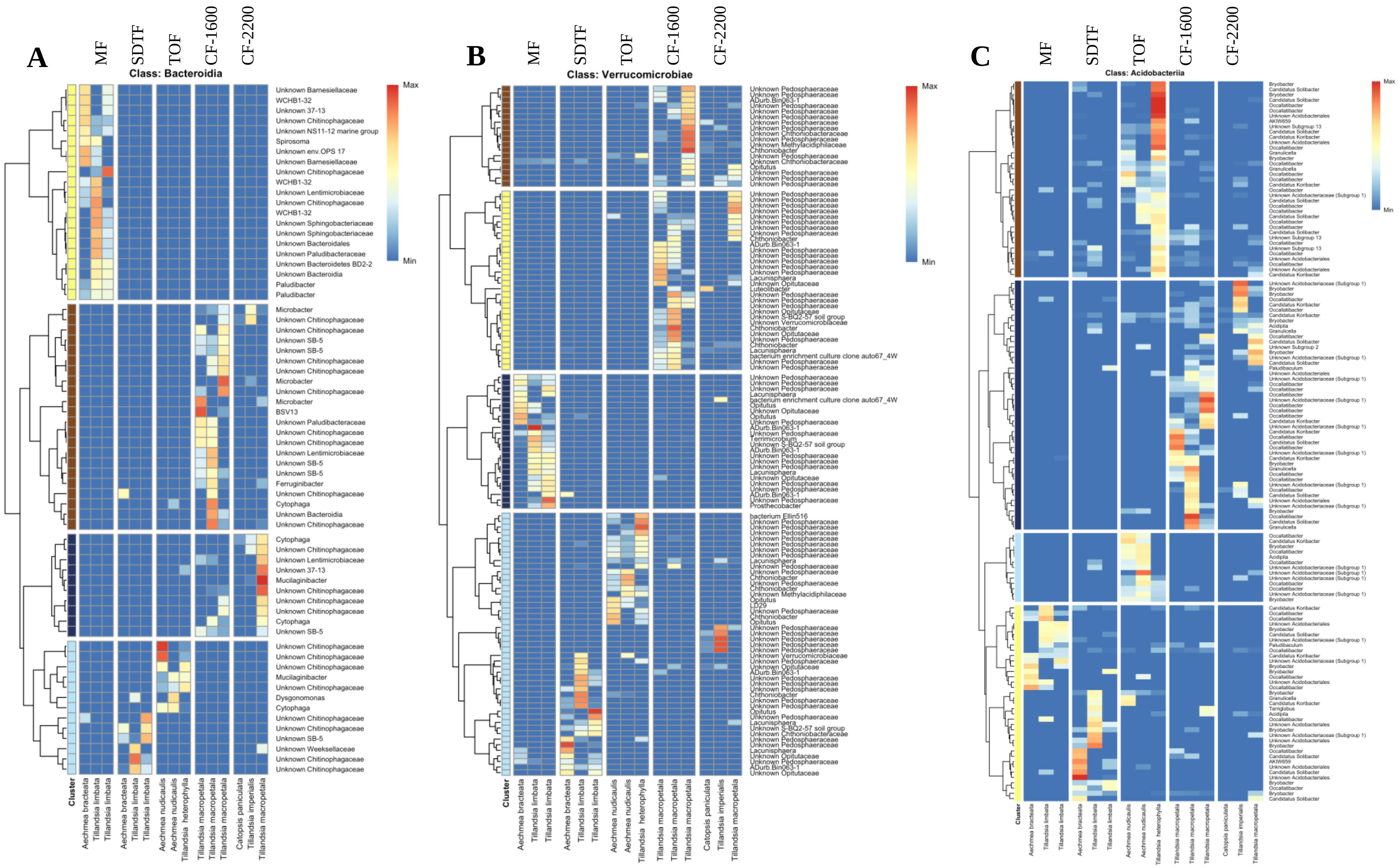

Figure S3. Heatmaps showing the composition of the classes Bacteroidia (A), Verrucomicrobiae (B) and Acidobacteriia (C) within the dataset. Mangrove forest (MF), semi-deciduous tropical forest (SDTF), tropical oak forest (TOF) and cloud forest (CF).
